# The use of tourniquet in total knee arthroplasty does not impact the functional outcome: a randomised controlled study

**DOI:** 10.1186/s13018-024-05203-y

**Published:** 2024-10-30

**Authors:** Magnus Johnsen, Steinar Havik, Vigdis Schnell Husby, Siri Bjørgen Winther, Olav A. Foss, Otto Schnell Husby, Øystein Bjerkestrand Lian

**Affiliations:** 1 Orthopaedic Department, Trondheim University Hospital, Post-box 3250 Torgarden, Trondheim, 7006 Norway; 2 Department of Health Sciences Aalesund, Faculty of Medicine and Health Science, Norwegian University of Science and Technology, Post-box 1517, Aalesund, NO-6025 Norway; 3 Department of Neuromedicine and Movement Science, Faculty of Medicine and Health Science, Norwegian University of Science and Technology, Post-box 8905, Trondheim, NO-7491 Norway; 4 Department of Orthopaedic Surgery, Kristiansund Hospital, Herman Døhlens vei 1, Kristiansund, 6518 Norway

**Keywords:** Total knee arthroplasty, Tourniquet, Functional outcome, Blood loss, Pain, Strength

## Abstract

**Background:**

This study evaluates the clinical evidence for performing total knee arthroplasty (TKA) without a tourniquet, a shift from the near-universal use in 2009 to current trends towards tourniquet-less TKA in Norway and Sweden. This change is set against a backdrop of conflicting evidence regarding the positive and negative effects of tourniquet use.

**Questions/purposes:**

The aims were to determine if the tourniquet has an impact on [1] Forgotten Joint Score-12 (FJS-12) at 8 weeks after surgery; [2] postoperative strength and function; [3] postoperative pain and opioid analgesic use; and [4] operative time, bleeding, and length of stay (LOS).

**Methods:**

Eighty-one patients were randomised to TKA with or without a tourniquet. The outcome measures, FJS-12, muscle strength, functional test, pain, estimated blood loss, haemoglobin (Hb) loss, knee circumference, opioid consumption, and LOS were assessed preoperatively and at 1 day, 8 weeks, and 1 year after surgery.

**Results:**

No significant difference in FJS-12 scores was found between the two groups at postoperative 8 weeks. However, the tourniquet group showed statistically significant better knee extension strength at 8 weeks (*p* = 0.045). There were no differences in other outcomes, except for a greater decrease in haemoglobin levels (*p* = 0.02) and higher estimated perioperative blood loss (*p* < 0.001) in the no tourniquet group than the torniquet group.

**Conclusions:**

Our study indicates that tourniquet use during TKA causes no significant differences in FJS-12 at 8 weeks, significantly reduces bleeding and postoperative Hb loss, and improves quadriceps strength at 8 weeks.

**Trial registration:**

Clinicaltrails.gov. Registry Number: NCT03666598. Registered 30 August 2018.

## Introduction

### Background

During total knee arthroplasty (TKA), a pneumatic tourniquet is often used to achieve a bloodless field. This likely enhances visualisation, optimises cementation, and reduces operating time [1, 2]. However, the use of a tourniquet has been associated with several potential negative effects such as reduced quadriceps strength [3, 4] and range of motion (ROM) [5–7] and increased tissue swelling [7] and pain [2, 7–9]. Furthermore, the use of a tourniquet may lead to adverse effects such as thromboembolic events [5, 8] and peripheral nerve injury [10].

While there is a general consensus that tourniquet use reduces intraoperative blood loss [7, 11–14], some studies indicate that the postoperative (hidden) blood loss increases, such that the total blood loss may be the same or even higher when using a tourniquet [7, 12, 14].

A survey conducted at the 2009 Annual Meeting of the American Association of Hip and Knee Surgeons (AAHKS) showed that almost all surgeons used some form of tourniquet during TKA [15]. Since then, a number of randomised clinical trials have been published showing conflicting evidence regarding the positive and negative effects of tourniquet use during TKA [7–9, 11, 16, 17]. In parallel with the ongoing debate, there has been a trend towards performing TKA without the use of a tourniquet. In 2022, 7,785 primary TKAs were performed in Norway, and 40% of them were without the use of a tourniquet [18]. According to the Swedish Arthroplasty Register, more than 72% of the approximately 17,000 TKAs performed in 2022 was done without the use of a tourniquet [19].

The aim of our study was to examine the clinical evidence supporting the trend towards performing TKA without the use of a tourniquet. In addition to assessing the patient’s subjective feedback, we wanted to examine a wide range of objective measures. The present randomised controlled trial, with (1) difference between the groups in Forgotten Joint Score-12 (FJS-12) 8 weeks after surgery as the primary outcome, also assessed the impact on factors such as (2) postoperative knee strength and function, (3) postoperative pain and opioid analgesic use and (4) operative time, bleeding volume, and length of stay.

## Method and patients

### Study design

This double-blind randomised controlled study was conducted by a team of researchers at the Department of Orthopaedic Surgery, Trondheim University Hospital. The patients were recruited from the outpatient clinic by research associates between June 2019 and September 2020. The study was conducted in accordance with the Declaration of Helsinki and approved by the Regional Committee for Medical and Health Research Ethics (REK 2018/42). Written informed consent was obtained from all patients. The study was registered at Clinicaltrials.gov (NCT03666598) prior to patient inclusion and reported according to the CONSORT guidelines.

### Participants

**Fig. 1:**
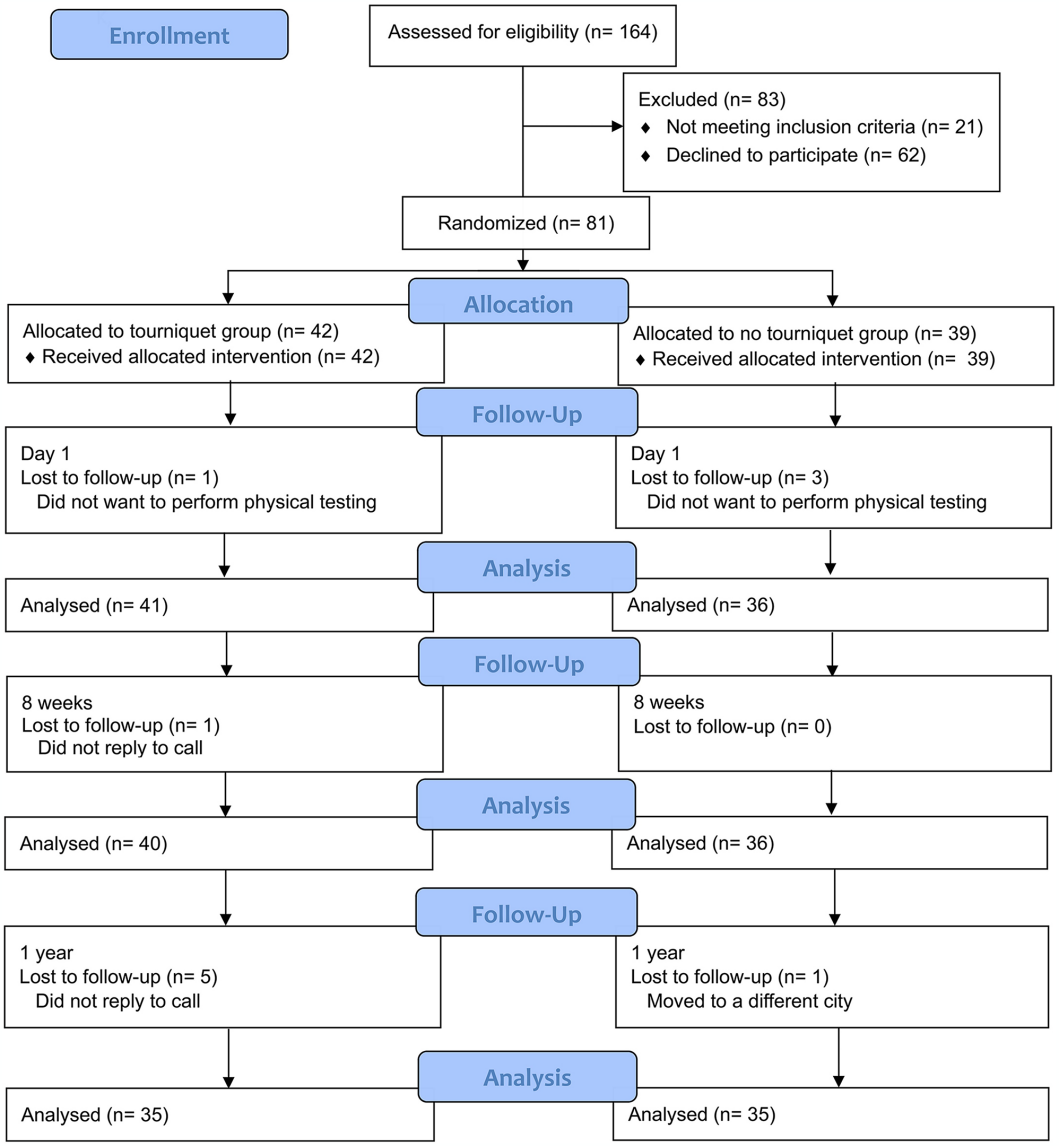
CONSORT flow diagram of patient enrolment and analysis

Patients were eligible for inclusion if they were diagnosed with osteoarthritis qualifying for TKA. The exclusion criteria were age < 18 years, coagulopathy, rheumatoid arthritis, peripheral vascular disease, malignancy, ongoing infection, contralateral gonarthrosis in need of treatment, and the inability to understand verbal and written information in Norwegian. The CONSORT flow chart is shown in Fig. 1. Patient demographics are presented in Table 1.

**Table 1:** Demographic data of the patients

		Tourniquet	No tourniquet	T-NoT (95% CI)
Sex	Male	14 (17.3%)	12 (14.8%)	
	Female	28 (34.6%)	27 (33.3%)	
Age (years)		66 (11)	68 (7)	-2 (-5.4 to 3.2)
BMI (kg/m^2^)		30 (6)	29 (4)	1 (-1.7 to 3.1)

T = Tourniquet group; NoT = No tourniquet group; CI = Confidence interval

Mean values (SD) and group differences (95% CI)

### Randomisation

The patients were randomly assigned in a 1:1 ratio to undergo surgery with or without tourniquet via a computer-generated list (Unit for Applied Clinical Research, Norwegian University of Science and Technology, Norway) and stratified according to sex. All patients had a pneumatic tourniquet cuff on the proximal thigh to ensure blinding, but only those randomised to the tourniquet group had the tourniquet inflated. The randomisation result was shown to the surgeon immediately before surgery. The patients and research assistants were also blinded to group allocation.

### Surgical procedure

The surgical procedures were performed by two senior orthopaedic surgeons with extensive experience in TKA, both with and without the use of tourniquet. For infection prophylaxis, four doses of cefazolin 2 g (or clindamycin 600 mg in case of penicillin contraindication) were administered with a 180-min interval, with the first dose given 30–60 min before the start of surgery. Tranexamic acid 15 mg/kg (maximum 1.5 g) was administered intravenously at the start of the procedure. For all patients in the tourniquet group, the pneumatic tourniquet cuff was inflated to 300 mmHg before skin incision and was deflated before wound closure (Zimmer A.T.S. 750, Zimmer Biomet, Warsaw, IN, US). The surgery was performed through a standard medial parapatellar approach. All patients received the Persona (Zimmer Biomet, Switzerland) cemented cruciate-retaining TKA without patellar resurfacing. Bone resections and implant insertion were conducted according to the manufacturer’s manual. A local anaesthetic (150 ml naropin 2 mg/mL) was injected behind the joint capsule, alongside the collateral ligaments and the skin incision. After the capsule was closed, 2 g tranexamic acid mixed with 50 mL saline was injected into the joint. After wound closure, a compression dressing (Dana Universal) was applied, and the knee was placed in a flexed position until the patient left the recovery unit. Surgical and tourniquet times were recorded. Perioperative blood loss was estimated from sponges and suction drain reservoirs.

### Postoperative follow-up

All patients followed the standardised fast-track patient course at our hospital’s orthopaedic department [20]. Patients received a standard postoperative analgesia regime of paracetamol 1 g q.i.d. naproxen/esomeprazole 1 tablet b.i.d., tapentadol 50 mg b.i.d. and oxycodone 5 mg (if needed). Anticoagulation (enoxaparin 40 mg ×1 s.c.) was given until discharge. All patients had inpatient physiotherapy while admitted, and outpatient physiotherapy for 8 weeks. After 8 weeks all patients were assessed by a physiotherapist at the hospital.

### Outcomes

The primary outcome—FJS-12—was assessed at the outpatient clinic 8 weeks after surgery. Secondary outcomes were FJS-12, 1 repetition maximum (1RM) leg press, 1RM knee extension, active range of motion (AROM), stair climb test (SCT), numeric pain rating scale (NPRS), blood loss volume, haemoglobin (Hb) fall, knee circumference, morphine milligram equivalents (MME), and length of hospital stay (LOS). Assessments for the secondary outcomes were performed at the outpatient clinic preoperatively, on Day 1, and at 8 weeks and 1 year postoperatively.

### Data collection

FJS-12 measures the patients’ awareness of the joint and ranges from 0 to 100, where a higher value indicates a lower awareness of the joint in daily activities [21]. 1RM leg press was tested using a leg press ergometer with the patient in a supine position (Steens Physical; Ring Mekanikk, Moelv, Norway). The test was valid if the patient was able to perform the leg press movement from an extended position to flexion with a knee joint angle of 90° and return to the extended position. The weight was increased by 10 kg for each repetition, and the test was terminated when the participant was unable to perform the required movement. 1RM knee extension was tested using knee extension equipment (Body-Solid, Forest Park, IL, USA) with the patent in a seated position. The test was valid if the patient was able to fully extend their knee joint from a 90° angle. The weight was increased by 2.5–5 kg for each repetition, and the test was terminated when the patient was unable to perform the required movement. The minimum load lifted with this equipment was 2.5 kg. AROM was measured using a plastic goniometer [22]. For active knee flexion, the patients were instructed to maximally flex their knee. When measuring active knee extension, the patients were asked to maximally extend their knee. Negative values of knee extension represent extension deficits. SCT measured the time to ascend, turn, and descend a stairway of 11 steps. The patients were asked to perform the test as quickly as possible [23]. The level of pain experienced by the patients was assessed using NPRS. Patients were asked to rate their knee pain at rest and during activity on a scale from 0 to 10, where 0 indicates no pain and 10 indicates the worst pain imaginable. Perioperative blood loss was estimated during surgery, and pre- and postoperative Hb levels were measured. Knee circumference was measured to assess swelling. The patient was placed in a supine position and a tape measure was used to measure the circumference of the knee, 1 cm proximal to the patellar base [22]. To assess the patients’ opioid use, MME was calculated during admission using conversion factors described by the Centers for Disease Control and Prevention [24]. Data on LOS was recorded on the date of discharge.

### Statistical analysis

The primary outcome was the difference in FJS-12 between the two groups reported 8 weeks after surgery. No formal statistical sample size calculation was made. We were unable to identify any pervious publications reporting FJS-12 results for tourniquet use in TKA that could be used for sample size calculations. Consequently, the sample size was estimated based on Ejaz et al.’s study [25], which used the Knee Injury and Osteoarthritis Outcome Score (KOOS) questionnaire and finally included 70 patients. The FJS-12 does not have the ceiling effect that is seen in KOOS [26]; therefore, we expected larger differences between the groups in the present study. To account for dropouts, 40 patients were included in each group. Normally distributed continuous variables were presented using means (standard deviation [SD]). Non-normal variables were reported as median (interquartile range [IQR]). Visual inspection of histograms was used to inspect the data distribution. Differences in mean values between groups were compared using independent samples *t*-tests for normally distributed data and Mann–Whitney U-test for non-normally distributed data. For all statistical analysis, p *≤* 0.05 was considered statistically significant. Lines in graphs represent means and corresponding 95% confidence intervals (CIs). All statistical analysis were performed using IBM SPSS Statistics for Windows, version 29 (Armonk, NY, IBM Corporation).

## Results

### FJS-12 scores

**Fig. 2:**
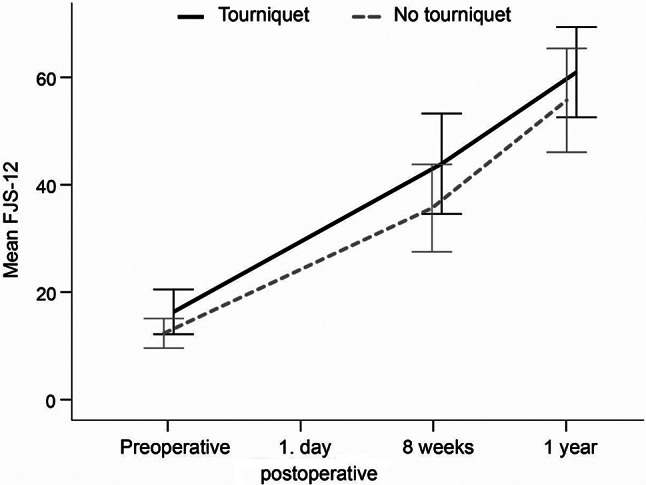
Forgotten Joint Score-12 (FJS-12) scores at different time points for each group

No statistically significant difference was observed in the FJS-12 8 weeks after surgery between the with and without tourniquet groups (44 *±* 28 vs. 36 *±* 24, mean difference (MD): 8, 95%CI: -1 to 9, *p* = 0.18). Detailed results on FJS-12 are presented in Table 2; Fig. 2.

**Table 2:** Mean values (SD), group differences (95% CI) and p-values for primary outcome and secondary outcomes

Variable	Preoperative			1. day postoperative			8 weeks			1 year		
	T	NoT	NoT-T (95% CI) *p*-values	T	NoT	NoT-T (95% CI) *p*-values	T	NoT	NoT-T (95% CI) *p*-values	T	NoT	NoT-T (95% CI) *p*-values
Forgotten joint score (0-100)	16 (13)	12 (8)	4 (-1 to 9) *p* = 0.12	.	.	.	44 (28)	36 (24)	8 (-1 to 9) *p* = 0.18	61 (25)	56 (29)	5 (-7 to 18) *p* = 0.41
1RM leg press (kg)	67 (23)	67 (26)	0 (-10.5 to 11.3) *p* = 0.94	26 (18)	28 (20)	-2 (-10.9 to 6.9) *p* = 0.65	64 (24)	61 (22)	3 (-7.6 to 13.5) *p* = 0.58	70 (24)	63 (31)	7 (-5.3 to 21.2) *p* = 0.24
1RM knee extension (kg)	14 (17)	13 (8)	1 (-5.1 to 6.5) *p* = 0.80	1 (2)	0 (1)	1 (-0.1 to 1.2) *p* = 0.13	16 (15)	10 (6)	6 (0.1 to 10.8) *p = 0.045	20 (14)	20 (17)	0 (-7.2 to 7.7) *p* = 0.95
Active knee flexion (°)	112 (11)	115 (12)	-3 (-8.3 to 1.9) *p* = 0.22	81 (11)	81 (11)	0 (-5 to 5.2) *p* = 0.96	107 (10)	111 (12)	-4 (-9.4 to 0.7) *p* = 0.09	110 (9)	111 (11)	-1 (-5.6 to 4.1) *p* = 0.75
Active knee extension (°)	-8 (6)	-7 (6)	-1 (-4.1 to 1.1) *p* = 0.26	-19 (11)	-17 (9)	-2 (-6.4 to 2.7) *p* = 0.42	-6 (5)	-7 (5)	1 (-1.8 to 2.8) *p* = 0.65	-3 (4)	-3 (4)	0 (-1.7 to 1.9) *p* = 0.9
Stair Climb test (sec)	18.8 (12.5)	18.3 (12.1)	0.5 (-4.9 to 6.0) *p* = 0.84	50.0 (25.0)	49.7 (26.9)	0.3 (-12.1 to 12.8) *p* = 0.96	16.6 (8.8)	18.8 (12.7)	-2.2 (-7.2 to 2.8) *p* = 0.38	12.7 (6.0)	15.9 (12.3)	-3.2 (-7.9 to 1.4) *p* = 0.17
Knee circumference (cm)	40.1 (5)	39.1 (4)	1 (-0.9 to 3.01) *p* = 0.29	42.6 (4.2)	43.0 (3.5)	-0.4 (-2.1 to 1.5) *p* = 0.72	.	.	.	.	.	.
NPRS rest (0–10)	3 (2)	3 (3)	0 (-2 to 0.5) *p* = 0.35	3 (2)	3 (2)	0 (-0.8 to 1.3) *p* = 0.63	1 (1)	1 (2)	0 (-1.1 to 0.6) *p* = 0.52	0 (1)	1 (2)	-1 (-1.0 to 0.3) *p* = 0.29
NPRS activity (0–10)	6 (2)	6 (2)	0 (-0.8 to 1.1) *p* = 0.78	5 (2)	5 (2)	0 (-1.2 to 0.9) *p* = 0.77	2 (2)	2 (2)	0 (-1.3 to 0.4) *p* = 0.28	2 (2)	2 (2)	0 (-1.7 to 0.5) *p* = 0.27

*Significant group difference. T = Tourniquet group; NoT = No tourniquet group; CI = Confidence interval. NPRS; Numeric Pain Rating Scale, where 0 is no pain and 10 is the worst pain imaginable. Forgotten Joint Score; 0-100, where 100 is the highest score

### Postoperative strength and function

**Fig. 3:**
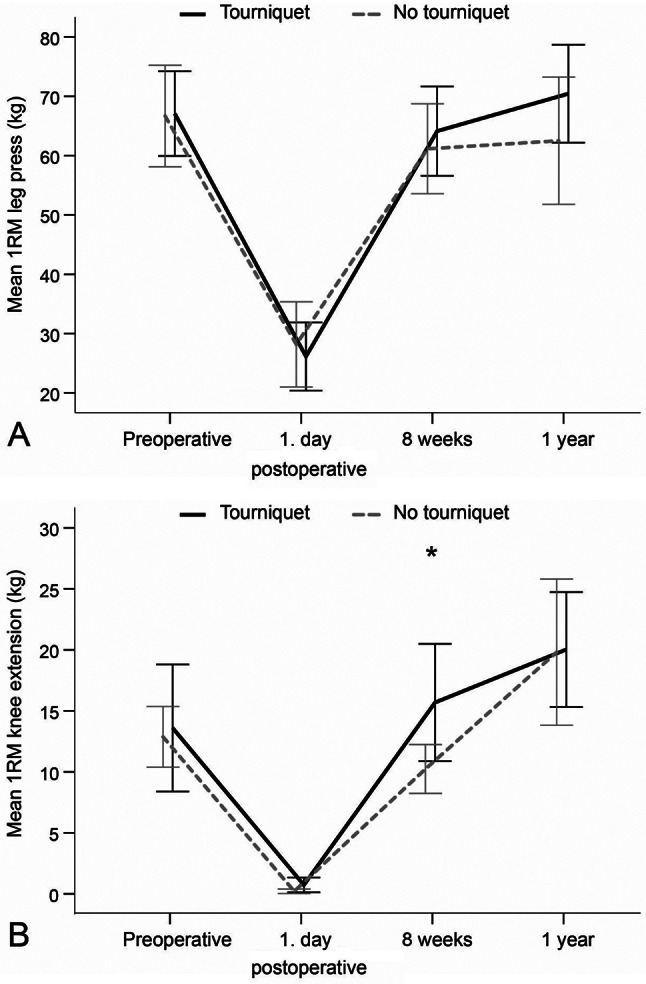
**A** and **B**. 1 repetitive maximum (RM) leg press and 1RM knee extension at different time points for each group. *Statistically significant difference

**Fig. 4:**
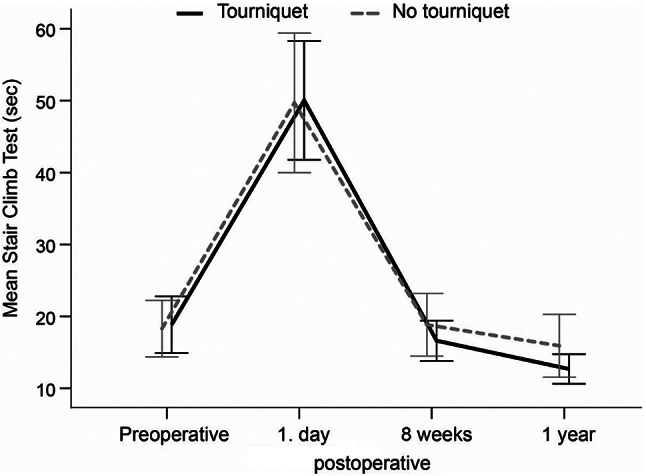
Stair climb test (SCT) results at different time points for each group

**Fig. 5:**
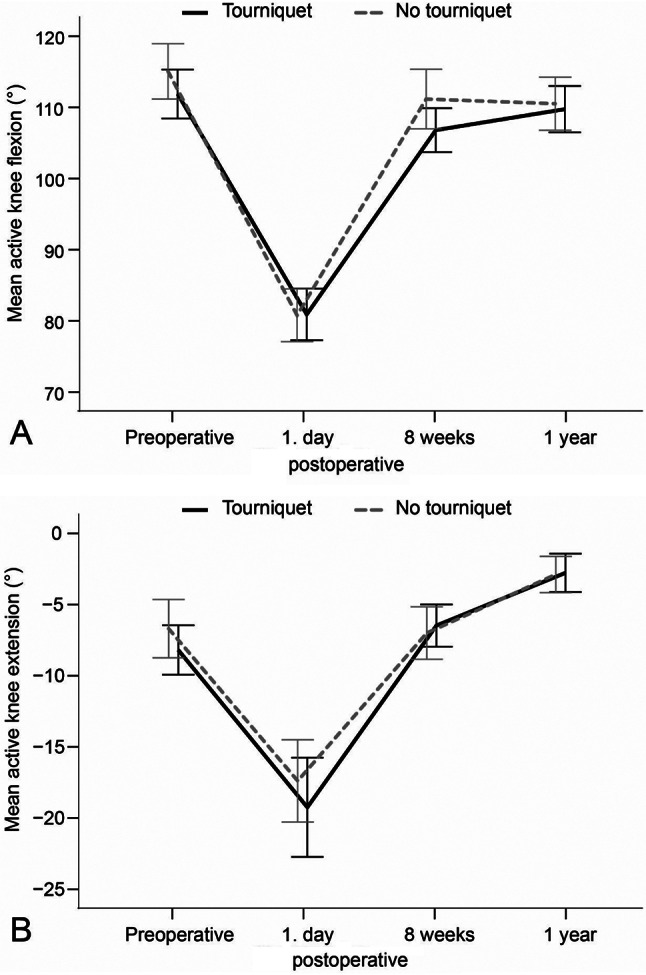
**A** and **B**. Active knee flexion and extension at different time points for each group

No statistically significant intergroup differences were found with regard to 1RM leg press at any of the assessment times (Table 2; Fig. 3A). In the assessment of 1RM knee extension, the tourniquet group lifted significantly heavier weights than the no tourniquet group 8 weeks after surgery (16 *±* 15 vs. 10 *±* 6, MD: 6, 95%CI: 0.1–10.8, *p* = 0.045) (Table 2). No significant differences were observed at the other assessment times (Fig. 3B). No statistically significant intergroup differences were found with respect to SCT, AROM, or knee circumference at any of the assessment times (Table 2; Figs. 4 and 5A, and 5B).

### Postoperative pain and opioid analgesic use

**Fig. 6:**
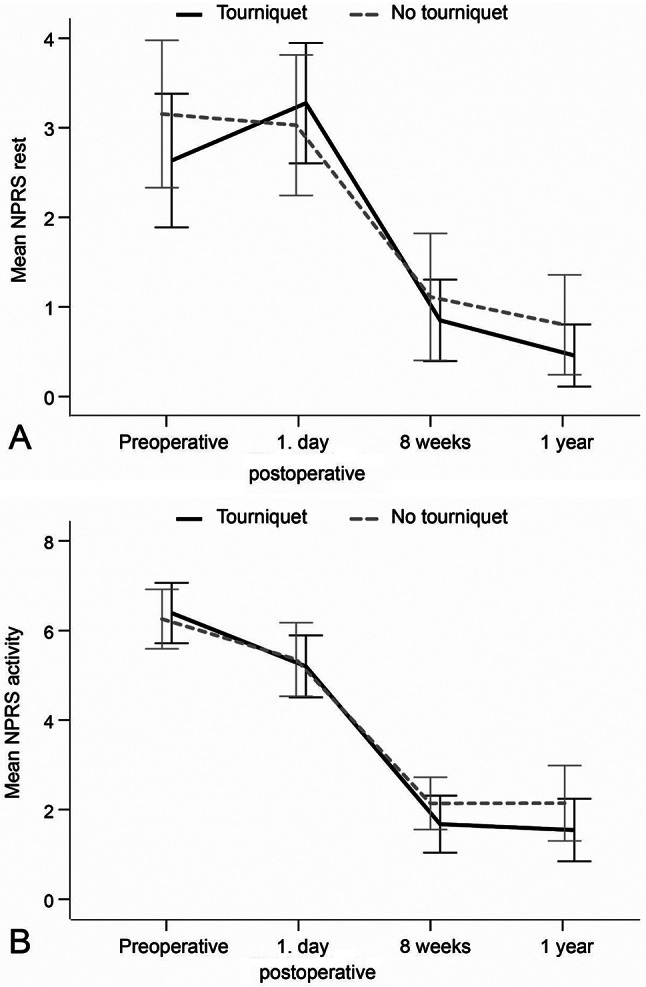
**A** and **B**. Numeric pain rating scale (NPRS) scores during rest and activity at different time points for each group

There were no statistically significant differences in NPRS between the groups at any of the assessed time points, either at rest or during activity (Table 2; Fig. 6A and B). There were no statistically significant differences in MME 1 day postoperatively between the tourniquet and no tourniquet groups (23.3 *±* 26.8 vs. 23.6 *±* 22.6, MD: -0.3, 95%CI: -12.0 to 11.3, *p* = 0.955).

### Operative time, bleeding and length of stay

There were no statistically significant differences in operative time between the groups (Table 3). The no tourniquet group had a significantly greater fall in Hb levels from pre- to postoperative assessments (1.7 *±* 0.6 vs. 2.1 *±* 0.9, MD: -0.4, 95%CI: -0.8 to -0.1, *p* = 0.02) (Table 3). The no tourniquet group had a significantly higher perioperative estimated blood loss volume than the tourniquet group (122 *±* 136 vs. 254 *±* 143, MD: -132, 95%CI: -196 to 69, *p* < 0.001) (Table 3). No significant difference in LOS was found between the two groups (1.92 *±* 0.53 vs. 1.97 *±* 0.62, MD: -0.05, 95%CI: -0.31 to 0.21, *p* = 0.70).

**Table 3:** Perioperative measures, length of stay

	Tourniquet		No tourniquet		T-NoT (95% CI) *p*-value
	Median (IQR)	Mean (SD)	Median (IQR)	Mean (SD)	Difference in mean/median
Operative time (min)	78 (19)		71 (15)		7 *p* = 0.059
Tourniquet time (min)	60.5 (17)		.		.
Hb fall (g/dL)		1.7 (0.6)		2.1 (0.9)	-0.4 (-0.8 to -0.1) *p = 0.02
Estimated blood loss (ml)		122 (136)		254 (143)	-132 (-196 to 69) *p = < 0.001
Length of stay (days)		1.92 (0.53)		1.97 (0.62)	-0.05 (-0.31 to 0.21) *p* = 0.70

*Significant group difference. T = Tourniquet group; NoT = No tourniquet group; CI = Confidence interval

### Adverse events

One patient in the tourniquet group was diagnosed with deep venous thrombosis 4 weeks postoperatively. One patient from each group had to undergo mobilisation under anaesthesia due to persistent stiffness of the operated knee joint.

## Discussion

The main finding of this study was that using a tourniquet during TKA does not cause a statistically significant difference in FJS-12 at 8 weeks after surgery compared to not using a tourniquet. Additionally, the tourniquet group had statistically significant better quadriceps strength than the no tourniquet group 8 weeks after surgery, as well as a statistically significant lower perioperative blood loss and less fall in postoperatively Hb levels. There were no other significant intergroup differences when looking at the other variables.

### FJS-12 score

No significant difference in FJS-12 was found between the groups 8 weeks after surgery. However, the tourniquet group had a higher mean score at all evaluations than the no tourniquet group. The FJS-12 assesses patients’ ability to forget their artificial joint in daily life. Compared to the Western Ontario and McMaster Universities Arthritis Index-Osteoarthritis (WOMAC-OA) index and other traditional Patient Reported Outcome Measures (PROMs), the FJS-12 is superior in discerning patient outcomes owing to a considerably low ceiling effect [27, 28]. In a recent systematic review [29], nine studies on patient-reported knee function scores following TKA with and without tourniquet use were evaluated. A significant difference was found between the two groups in only one of the nine studies, where the tourniquet group scored worse after 3 months than the no tourniquet group. In another of nine studies, the tourniquet group had significantly higher scores after 2 months than the no tourniquet group. The result of our study are consistent with those of previous publications that found no significant differences between the groups.

### Postoperative strength and function

No significant difference in ROM was found between the tourniquet and no tourniquet groups at 1 day, 8 weeks, or 1 year after surgery. The tourniquet group had statistically significant better quadriceps strength than the no tourniquet group 8 weeks after surgery. However, at the 1-year follow-up, there was almost no difference between the two groups. Moreover, there were also no significant differences in leg press strength or stair climbing time at any of the follow-up time points between the groups.

These findings are in accordance with a systematic review and meta-analysis [16] which reported no clinically important differences in ROM between patients treated with or without a tourniquet. Furthermore, another systematic review [30] found that only two out of 17 studies reported a statistically significant difference in functional outcome. However, other meta-analyses [7, 31] concluded that tourniquet application was associated with significantly lower ROM and poorer knee function than without. Previous publications report increased postoperative knee swelling between 1 and 5 days after surgery when using a tourniquet. This has been attributed to a period of hyperaemia and fibrinolysis, which cause bleeding into adjacent soft tissue following the deflation of the tourniquet [13, 32]. No significant differences were observed between the groups when measuring knee circumference 1 day and 8 weeks after surgery. However, given that swelling often is highest between 1 and 5 days after surgery, it is possible that our assessments do not fully reflect the entire truth.

### Postoperative pain and opioid analgesic use

No significant difference in pain at rest or during activity was found between the groups. Furthermore, there were no significant differences between the two groups in opioid use on the first postoperative day. Our results are consistent with several systematic reviews and meta-analyses [30, 33] that found no significant difference in patient-reported postoperative pain between the two groups. However, other systematic reviews and meta-analyses [8, 9, 29] reported contrary findings. Previous studies [34, 35] have reported higher opioid use in the tourniquet group than the no tourniquet group during the first postoperative day. This contrasts with our findings. Our results might have been influenced by the use of a multimodal pain management regime [20], which could have contributed to reducing the potential pain-aggravating effects of a perioperative tourniquet in the early postoperative phase.

### Operative time, bleeding volume, and LOS

Our study showed no significant difference in operating time between the tourniquet and no tourniquet groups. It has been suggested that the bloodless field created by a tourniquet improves visualisation during surgery, which in turn reduces operating time. This theory was not supported by our results. However, other authors have reported different conclusions. A meta-analysis [11] found that operating time was significantly shorter in the full-time tourniquet group than in the no tourniquet group. Another meta-analysis concluded that tourniquet use was associated with a shorter length of surgery than the group without a tourniquet [8]. When comparing blood loss between the two groups, we found that the no tourniquet group had a significantly greater drop in Hb levels from the pre- to post-operative period with a significantly higher estimated perioperative blood loss than the tourniquet group. This is consistent with other studies [11]. However, several publications state that the use of a tourniquet reduces perioperative blood loss, but it does not reduce the total blood loss [12, 29, 36]. Total blood loss is based on both visible and hidden blood loss, and there are numerous publications that indicate that hidden blood loss is increased with the use of a tourniquet [13, 36, 37]. Hidden blood loss is defined as total blood loss minus perioperative and postoperative blood loss and is the continuous blood loss after surgery [36, 38]. In addition, the highest blood loss is reported to occur between 3 and 7 days after surgery [38, 39]. We did not assess the total blood loss, and we measured the Hb levels 1 day after surgery, so our results regarding blood loss may be interpreted by caution. In our study, there was no significant difference in LOS between the tourniquet and no tourniquet groups. This result was expected as all patients were enrolled via the same standardised fast track pathway. Our results contradict the findings of two meta-analysis [7, 11], which concluded that the use of a tourniquet resulted in a longer hospital stay.

### Limitations

In our study, the surgeons were experienced and the overall tourniquet and operative times were short. Longer tourniquet times have been described to have a negative effect on postoperative recovery [34]. Therefore, our results cannot be extrapolated to procedures with longer tourniquet times. Additionally, the absence of patient-reported pain assessment between 1 day and 8 weeks after surgery may have resulted in failure to identify potential group differences in the early postoperative period. Furthermore, although a common practice in estimating perioperative blood loss in the clinical setting, the estimated perioperative blood loss may lack precision as it was estimated by visual inspection by the anaesthetist nurse from sponges and suction drain reservoir. Last, the surgery was performed using a standard medial parapatellar approach. Therefore, the results of this study may not be generalisable to other approaches used in TKA.

## Conclusion

Our study found no clinically significant differences between the groups with respect to FJS-12 8 weeks after surgery. Use of tourniquet led to significantly less bleeding during surgery and a significantly lower fall in postoperative Hb levels. Furthermore, the tourniquet group demonstrated statistically significant better quadriceps strength than the no tourniquet group 8 weeks after surgery.

## Data Availability

No datasets were generated or analysed during the current study.

## Abbreviations

1RM: 1 Repetition Maximum
AROM: Active Range Of Motion
FJS-12: Forgotten Joint Score-12
Hb: Haemoglobin
KOOS: Knee Injury and Osteoarthritis Outcome Score
LOS: Length Of Stay
MME: Morphine Milligram Equivalents
NPRS: Numeric Pain Rating Score
ROM: Range Of Motion
SCT: Stair Climb Test
TKA: Total Knee Arthroplasty
